# Sexual violence as a predictor of unintended pregnancy among married women of India: evidence from the fourth round of the National Family Health Survey (2015–16)

**DOI:** 10.1186/s12884-022-04673-4

**Published:** 2022-04-21

**Authors:** Priyanka Garg, Madhur Verma, Priyanka Sharma, Carolina V. N. Coll, Milan Das

**Affiliations:** 1 Department of Obstetrics and Gynaecology, All India Institute of Medical Sciences, Bathinda, Punjab 151001 India; 2 Department of Community & Family Medicine, All India Institute of Medical Sciences, Bathinda, Punjab 151001 India; 3 Department of Community Medicine, North Delhi Municipal Corporation Medical College and Hindu Rao Hospital, New Delhi, 110007 India; 4 Postgraduate Program in Epidemiology, Federal University of Pelotas, Pelotas, Brazil; 5 Department of Population & Development , International Institute for Population Sciences (IIPS), Mumbai, Maharashtra 400088 India

**Keywords:** Sexual violence, Unintended pregnancy, Intimate partner violence, NFHS

## Abstract

**Background:**

Sexual Intimate Partner Violence (IPV) is a public health problem globally, with about one in three women experiencing sexual IPV ever in their lifetime. Unintended pregnancy is one of the consequences of sexual IPV and has its repercussions that can span generations. The present study was conducted to estimate the prevalence of sexual intimate partner violence (IPV) and assess the association between sexual IPV and unintended childbirth in India among married women aged 15–49 years.

**Methods:**

The National Family Health Survey-India (NFHS-4) fourth-round dataset was used for the present study. Pregnancies intention was the primary outcome variable, and the main predictor variable was self-reported sexual IPV in the past 12 months. Women’s current age, age at marriage, education and occupation, place of residence, wealth quintile, parity, religion, caste, region, mass media exposure, and husband’s education were other control variables. Weighted analysis depicted the prevalence of unintended pregnancies and their association with different socio-demographic variables. Binary logistic regression was done in two steps respecting a hierarchical approach for potential confounders.

**Results:**

Approximately 6.4% of study participants had ever experienced sexual IPV in India. Prevalence of sexual IPV was significantly higher when the age of marriage was < 19 years, among uneducated, in the lowest wealth index quintile, belonging to scheduled caste, having multiparity, and not having mass media exposure. About 12.1% of pregnancies were considered unintended by the respondents, and 22.9% of women who ever had a history of sexual IPV considered the last pregnancy to be unintentional. Women who experienced sexual IPV were in younger age groups, having parity ≥1, and bigger families had significantly higher odds of having an unintended pregnancy compared to their reference groups.

**Conclusions:**

We observed that sexual IPV has a significant role in unintended pregnancies. Effective counseling means should be rolled out for victims of sexual IPV as it is a taboo subject. The significant factors that can predict unintended pregnancies highlighted in our study should be acknowledged while counseling.

## Introduction

Sexual Intimate Partner Violence (IPV) is a grave human rights and public health problem worldwide [1]. Any sexual act or an attempt to obtain a sexual act, acts to traffic, or directed otherwise, against a person’s sexuality by coercion, any unwanted sexual advances or comments, by any person irrespective of their relationship to the victim, regardless of setting, including but not limited to work and home, falls under the aegis of sexual violence [2]. Across their lifetime, 1 in 3 women are subjected to sexual IPV. The lifetime prevalence of sexual Intimate Partner Violence (IPV) over the last 12 months has been estimated to be around 5.2% using the data from the fourth round of the National Family Health Survey (NFHS-4) [3]. This number has remained unchanged over the past decade [2, 4].

The risk factors for sexual IPV against women are young age, substance use, previous history of sexual abuse, having multiple sexual partners, involvement in sex work, and poverty in general, while being more educated and economically empowered is commonly a risk factor for IPV [2]. It has short-term and long-term repercussions on victims’ mental, sexual, physical, and reproductive health and is a highly distressing and violating experience for the survivor [1]. A woman, who has been a victim of sexual coercion at an early age, has reduced capability to discern her sexuality as something she can control. Rapes can also result in pregnancy, and the rates of such pregnancies vary with the extent of non-barrier contraceptive use [2]. Centres for Disease Control and Prevention defines an unintended pregnancy as unwanted or untimed [5]. In a recent study conducted in six south Asian countries, about one-fifth of total pregnancies were reported to be unintended, while it was 11.9% for India [6]. A frequent consequence of such pregnancies is an abortion, leading to long-term severe and adverse health effects like infertility and maternal death, especially in developing nations, due to poverty, malnutrition, inadequate sanitation, and low literacy levels [7]. The mothers were also less likely to receive antenatal care and supervised delivery [8]. A study from Bangladesh showed that neonatal and postnatal mortality rates were higher among unwanted children [8]. Further, the consequences of unintended pregnancy could result in stunting alongside the immediate negative effect on vaccination, most probably due to the disadvantage the unwanted children face [9].

The common reasons cited in literature from various countries for unintended pregnancies are poverty, lower educational status, inadequate access to health services, failure of family planning methods, and sexual violence [10, 11]. The role of established risk factors for unintended pregnancies in the victims of sexual IPV can be explained using the regression analysis in two steps respecting a hierarchical approach for potential confounders, where they may exert a complex hierarchical inter-relationships among themselves. Therefore, the decision to include risk factors in the final model is not just driven by the statistical significance but also on a conceptual framework that describes the theoretical hierarchy among the determinants. The risk factors are categorized as proximate and distal factors, and the approach ensures that the effect of distal factors is recognized even in the presence of proximate factors.

The critical policy decisions to mitigate unintended pregnancies are driven by robust evidence about the potential risk factors. However, literature is insufficient in India, and little is known about the effect of sexual IPV on unintended pregnancy in the country. Therefore, this secondary data analysis was done using a nationally representative dataset with the primary aim to estimate the prevalence of sexual IPV (ever history) amongst the married women aged between 15 and 49 years and assess the association between sexual IPV and unintended childbirth among currently married women of 15–49 years who experienced birth in last 5 years. The secondary objective was to look for other predictors of unintended pregnancy amongst the victims of sexual IPV informed by a two-step regression model.

## Methodology

### Source of data

We used the nationally representative National Family Health Survey-India (NFHS) dataset to fulfill this analysis’s objectives. The NFHS is part of the Global Demographic and Health Survey (DHS). The NFHS focus on maternal and child health, IPV, violence during pregnancies, contraception, and women’s empowerment. The fourth round of NFHS also asked about the frequency of domestic violence in the past 12 months. NFHS has employed a two-stage stratified sampling technique to collect the data. The details about NFHS sample techniques and procedures are mentioned elsewhere [12]. .Around 6,99,686 women in the reproductive age groups (15–49) were interviewed from 601,509 household samples from India’s states and union territories. The questions related to domestic violence have been asked in a separate module called the domestic violence module, which contained 15% of the samples from the state modules. The total sample size of the NFHS domestic module was 79,729.

### Sample selection

**Fig. 1:**
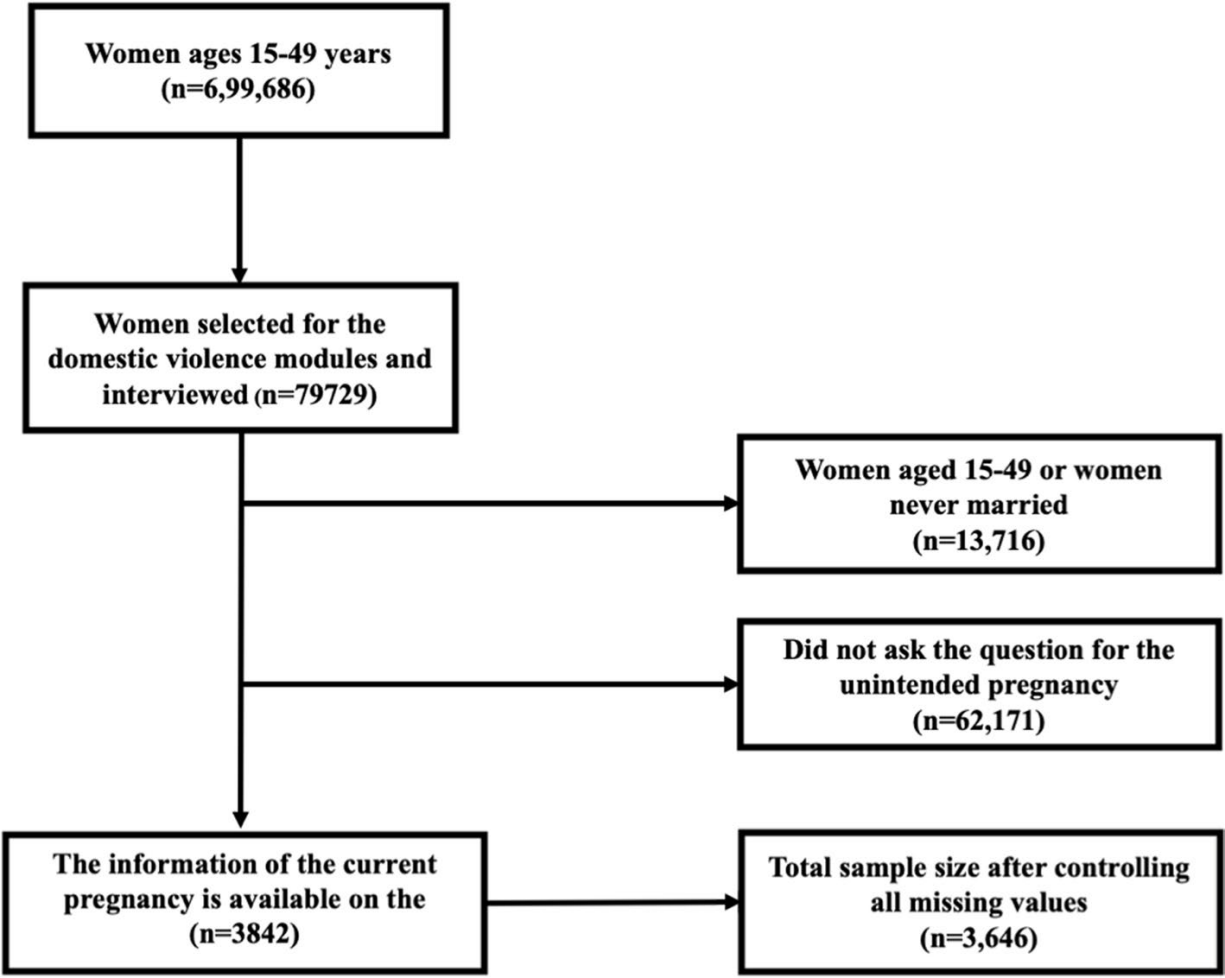
Flow chart depicting sample selection procedure

The women from the selected households were chosen for administering the domestic violence module. The unit of the analysis for this study was women aged 15–49 (*n* = 2045) who gave birth to at least one child in the last 5 years preceding the survey, and who are currently pregnant, and also provided information on fertility preference in relation after the child (Fig. 1). The respondents were randomly invited from the households of the state module to participate in the domestic violence module. If more than one woman in a home, only one woman was selected for this module.

### Dependent variable

Pregnancies intention was our primary outcome variable for the analysis. In the DHS, it was based on the question, “When you were pregnant, was the pregnancy wanted?” The responses were “then,” “later,” or “not at all.” Thus, women responding their last birth was ‘wanted later’ or ‘not wanted at all’ were characterized to have an unintended pregnancy, and those who answered ‘wanted birth then’ were considered to have an intended pregnancy. Unintended pregnancy was coded ‘1’ and intended pregnancy coded ‘0’.

### Predictor variable

The primary predictor variable in our analysis was self-reported sexual IPV ever experienced by the women. The DHS generate this data by asking: “Did you ever experience physical force by husband/partner to have sexual intercourse when you did not want to?”, Did your husband/partner use physical force to perform any other sexual acts when you did not want to?” and “Were you ever forced by your husband/partner with threats or in any other way to perform sexual acts when you did not want to?” " Likewise, the physical IPV was defined as "any type of physical violence experienced by a women at the hands of husband/partner, which includes: (a) ever having been slapped; (b) ever having had arm twisted or hair pulled; (c) ever having been pushed, shaken or had something thrown at them; (d) ever having been punched with fist or hit by something harmful; (e) ever having been kicked or dragged; (f) ever having been strangled or brunt; (g) ever having been threatened with knife/gun or other weapon. The answer to these questions were "never", "often", "sometimes," and "yes, but not in the last 12 months". If the answer was either “often”, “sometimes,” or “yes but not in the last 12 months” we considered the women to have ever experienced either Sexual or Physical IPV. For analysis we categorised type of IPV as "No IPV", "Only sexual IPV", "Only Physical IPV", and "Both Sexual and Physical IPV".

### Control variables

A thorough literature review was done, and control variables were considered because of their statistically significant relationship with pregnancy intentions. These variables included the age of the women (15–19,20-24,25–34 and 35 and above); Women’s age at marriage (marriage before aged 18, and marriage after age 18); place of residence (urban and rural); regions of India as per DHS; women’s and husband’s educational attainment (no education, primary, secondary and higher); wealth status (poorest, poorer, middle, richer, richest quintile); the religious belief of the households (Hindu, Muslim, Christian, Sikh, Others); caste (scheduled caste, Scheduled tribe and Others, and ‘other’ caste included general category and Other Backward Classes); parity (1 parity, 2 parities,3+ parities); and Exposure to mass media (watching television, watching radio, frequency of reading newspapers). Based on the women’s exposure to any of them, mass media exposures were classified as ‘yes’ or ‘no.’

### Statistical analysis

Analysis was done using STATA-14 software. We used bivariate analysis to examine the association between unintended pregnancy with the main predictor (sexual IPV) and control variables. Weighted analysis was done using the weights given for the domestic violence modules, i.e., d005. We further checked the association between sexual IPV with control variables. Chi-square testing was done to report *p*-values to check the hypothesis.

In the first stage, unadjusted odds ratios were calculated between the primary dependent variable, i.e., unintended pregnancy, and the primary independent variable, i.e., presence or absence of only sexual violence, only physical violence, both, or none. Then adjusted Odds ratio with 95% CI were calculated using a binary logistic regression analysis where the final model was constructed in two steps respecting a hierarchical approach for potential confounders. The first model included the sexual IPV along with the distal determinants. These included the socio-demographic variables like the age of the respondents, education, wealth, and place of residence as they affect pregnancy indirectly. The significant variables with a *p*-value < 0.2 were included in the second (final) model along with the proximal risk factors as these factors exert more effect on the women’s reproductive behavior. The second level variables included religion, caste, parity of the respondent, and her exposure to the mass media. A *p*-value of less than 0.05 was considered significant.

## Results

Around 51% of study participants (currently married and pregnant women) belonged to 25–49 years. Approximately 74.8% of women were living in rural areas. A higher proportion of women (26.3%) were uneducated than their husbands (16.3%). Nearly one-fourth of participants (23.7%) belong to the poorest quintile. Most women (48.8%) were primipara, while 34.6% were nulliparous. One-third of women (35.1%) had no media exposure. We found that 6.4% of the participant had ever experienced sexual IPV in India (Table 1). Prevalence of sexual IPV was significantly higher when the age of marriage was < 19 years (9.6% vs. 3.9%; *p*-value< 0.001), respondents and their husbands were not educated (10.3, 10.8%), in the lowest wealth index quintile(10.8%), belonging to schedule caste (8.3%), having multiparity (13.4%), not having mass media exposure (9.3%), and if she ever experienced physical IPV also (20.1%).

**Table 1:** Prevalence of ever experience of sexual violence among currently married pregnant women

Variables	N (%)	Sexual violence		
		No	Yes	***P***-value
**Total number**	**3646**	**3401**	**245**	
**Overall Prevalence**	100	92.6	6.4	
**Age**				0.287
15–24	1785 (49)	93.1	6.9	
25–49	1861 (51)	94.2	5.8	
**First cohabitation age**				< 0.001
< 19 years	1591 (43.6)	90.4	9.6	
> 19 years	2055 (56.4)	96.1	3.9	
**Place of residence**				< 0.01
Urban	917 (25.2)	96.4	3.6	
Rural	2729 (74.8)	92.5	7.6	
**Women Education**				< 0.001
No education	958 (26.3)	89.8	10.3	
Primary	478 (13.1)	92.2	7.8	
Secondary	1776 (48.7)	94.5	5.5	
Higher	434 (11.9)	98.4	1.6	
**Husband education**				< 0.001
No education	593 (16.3)	89.2	10.8	
Primary	497 (13.6)	87.1	12.9	
Secondary	2003 (54.9)	94.7	5.3	
Higher	553 (15.2)	98.1	1.9	
**Wealth Index**				< 0.001
Poorest	865 (23.7)	89.2	10.8	
Poor	858 (23.5)	91.5	8.5	
Middle	746 (20.5)	93.6	6.4	
Richer	631 (17.3)	97.5	2.5	
Richest	546 (15)	97.1	2.9	
**Religion**				0.77
Hindu	2677 (73.4)	93.6	6.4	
Muslim	537 (14.7)	93.4	6.6	
Christian	283 (7.8)	95.3	4.7	
Others	149 (4.1)	93.1	6.9	
**Caste**				0.001
Scheduled Caste & Schedule Tribes	1466 (40.2)	91.7	8.3	
Others	2180 (59.8)	94.5	5.5	
**Parity**				< 0.001
None	1260 (34.6)	95.7	4.3	
1	1778 (48.8)	93.9	6.1	
2+	608 (16.7)	86.6	13.4	
**Household members**				0.09
1–3 members	939 (25.8)	94.7	5.3	
4–6 members	1970 (54)	93.2	6.8	
7+ members	737 (20.2)	93.6	6.4	
**Mass media exposure**				< 0.001
No	1280 (35.1)	90.7	9.3	
Yes	2366 (64.9)	94.9	5.1	
**Ever experienced Physical violence**				
Never	2925	97.8	2.17	
Yes	721	79.90	20.1	

Overall, 12.1% of pregnant women considered their pregnancies unintended. Table 2 depicts the association of unintended pregnancy with different socio-demographic variables. The prevalence was significantly higher in women who only reported sexual IPV (22.9%) than those who only reported physical IPV (14.3%) and those who reported both sexual and physical forms of IPV. Other significant associations were seen with higher age, i.e., between 25 and 49 years (13.2%), lower age (< 19 years) of the first cohabitation (15.4%), rural residence (13.6%), no-education (15.3%), poorest wealth index (16.5%), Muslim religious belief (16.1%), reserved caste (12.3%), multi-parity (28.0%), the higher number (+ 7) of household members (14.4%), and no mass media exposure (17.3%).

**Table 2:** Prevalence of unintended pregnancy and total sample sizes by socioeconomic variables, India

Variables	Unintended pregnancy		***p*** value
	No	Yes	
Total Number (%)	3206 (87.9)	440 (12.1)	
**Type of IPV **			< 0.001
No IPV	89.4	10.5	
Only Sexual	77.1	22.9	
Only Physical	85.7	14.3	
Both Sexual & Physical	77.5	22.5	
**Age**			< 0.05
15–24 years	88.9	11.1	
25–49 years	86.8	13.2	
**First cohabitation age**			< 0.001
< 19 years	84.6	15.4	
> 19 years	90.6	9.4	
**Place of residence**			< 0.01
Urban	91.7	8.3	
Rural	86.4	13.6	
**Women Education**			< 0.01
No education	84.7	15.3	
Primary	85.0	15.0	
Secondary	88.7	11.3	
Higher	93.5	6.5	
**Husband education**			< 0.001
No education	81.1	18.9	
Primary	88.2	11.8	
Secondary	87.9	12.1	
Higher	93.5	6.5	
**Wealth Index**			< 0.01
Poorest	83.5	16.5	
Poor	86.0	14.0	
Middle	87.3	12.7	
Richer	90.6	9.4	
Richest	93.1	6.9	
**Religion**			< 0.01
Hindu	88.7	11.3	
Muslim	83.9	16.1	
Christian	95.0	5.0	
Others	88.4	11.6	
**Caste**			< 0.01
Scheduled Caste and Tribes	87.7	12.3	
Others	88.0	12.0	
**Parity**			< 0.001
None	94.4	5.6	
1	87.0	13.0	
2+	72.0	28.0	
**Household members**			
1–3 members	92.1	7.9	
4–6 members	87.9	12.1	
7+ members	85.64	14.4	
**Mass media exposure**			
No	82.75	17.3	
Yes	90.28	9.7	

Table 3 shows the major risk factors for unintended pregnancies in victims of sexual violence that emerged following the two step regression model approach. The first model depicts the highest chances of having unintended pregnancies among the victims of sexual IPV (cOR: 2.72; 95% CI: 1.50–4.92) compared to the victims of only physical IPV (cOR: 1.55; 95% CI: 1.22–1.97), a victim of both sexual and physical IPV (cOR: 2.45; 95% CI: 1.69–3.56). The second model depicted sexual IPV, the older respondent age, and primary education as the significant predictors of unintended pregnancies. They were included in the final model along with the level 2 proximate independent variables. The adjusted OR of the violence variables depicted a similar pattern to the cOR, and sexual IPV was expressed as a more decisive risk factor for unintended pregnancies (OR: 2.90; 95% CI: 1.56–5.42), compared to physical IPV alone, or both physical and sexual IPV. Further, the final model depicted older age as a significant protective factor (OR: 0.72; 95% CI: 0.56–0.92), in contrast to the findings of the second model. Other significant factors were primary education, Christianism religion, primi or multiparity, and larger families. Social Caste, the first age of cohabitation, and media exposure depicted no influence on unintended pregnancies.

**Table 3:** Logistic regression estimates of the unintended pregnancy by socioeconomic characteristics, Indi

Variables	OR (95% CI)	OR (95% CI)	OR(95% CI)
**Type of IPV**			
No IPV	Ref.	Ref.	Ref.
Only Sexual	2.72***(1.50–4.92)	2.56***(1.40–4.65)	2.90***(1.56–5.42)
Only Physical	1.55***(1.22–1.97)	1.46***(1.14–1.87)	1.23 (0.96–1.58)
Both Sexual & Physical	2.45***(1.69–3.56)	2.30***(1.57–3.37)	1.88***(1.27–2.78)
**Age**			
15–24		**Ref.**	**Ref.**
25–49		1.30**(1.05–1.59)	0.72*(0.56–0.92)
**Place of residence**			
Urban		Ref.	–
Rural		1.26 (0.95–1.66)	–
**Women Education**			
No education		**Ref.**	–
Primary		0.88 (0.63–1.25)	–
Secondary		0.90 (0.68–1.20)	–
Higher		0.91 (0.56–1.48)	–
**Husband education**			
No education		**Ref.**	**Ref.**
Primary		0.66*(0.45–0.96)	0.66*(0.45–0.97)
Secondary		0.96 (0.71–1.30)	1.09 (0.82–1.45)
Higher		0.71 (0.44–1.14)	0.92 (0.60–1.41)
**Wealth Index**			
Poorest		**Ref.**	–
Poor		1.03 (0.77–1.38)	–
Middle		1.01 (0.73–1.40)	–
Richer		0.91 (0.62–1.33)	–
Richest		0.80 (0.51–1.26)	–
**Religion**			
**Hindu**			**Ref.**
Muslim			1.02 (0.77–1.36)
Christian			0.62*(0.39–1.00)
Others			0.56 (0.28–1.13)
**Caste**			
Scheduled Caste and Tribes			**Ref.**
Others			1.22 (0.96–1.54)
**Parity**			
None			**Ref.**
1			2.83***(2.09–3.84)
2+			6.34***(4.24–9.47)
**First cohabitation age**			
< 19 years			**Ref.**
> 19 years			1.08 (0.86–1.36)
**Household members**			
1–3 members			**Ref.**
4–6 members			1.32 (0.99–1.76)
7+ members			1.71***(1.24–2.36)
**Mass media exposure**			
No			**Ref.**
Yes			0.86 (0.68–1.08)

## Discussion

The present study is among the few studies from India to provide national-level estimates on the effect of sexual IPV on unintended pregnancy. We investigated the association using data of 3646 married pregnant women and observed around 12% prevalence of unintended pregnancies in the victims of sexual IPV. Studies from Uttar Pradesh and the slum area of Mumbai have reported that 36 and 43% of the current pregnancies were unintended pregnancies [13, 14]. Previous sub-national estimates have reported the prevalence of unintended pregnancies in six states of India between 44 to 95 per 1000 women of reproductive age [15]. Another estimate from Bangladesh have reported the prevalence of unintended pregnancies to be around 31% [16]. In Nepal, Acharya et al. identified higher odds of unintended pregnancy among women whom their husbands had sexually abused [17]. Other studies from South Asian countries like Pakistan and Bangladesh have reiterated that sexual IPV influences mistimed and unwanted pregnancies [18–20]. The differences in the prevalence of unwanted pregnancies could be partly due to inclusion criteria and sample size variations between the studies. Also, the differences in the Total Fertility Rate and Couple Protection Rate of different study areas can affect the prevalence of unintended pregnancies. Another attributable factor for high prevalence is the male dominance in sexual decision-making over women’s wishes regarding fertility and contraceptive use [21, 22]. Surprisingly, an analysis from the previous rounds of DHS from South-Asian countries, including India observed that Sexual IPV was not associated with unintended pregnancies. This negative trend should be a cause of concern [23].

It was observed that women with a history of sexual IPV reported a higher risk of having unintended pregnancies, even when the socioeconomic and demographic characteristics of the respondents were controlled for through a hierarchical approach. The said higher odds of unintended pregnancy in victims of sexual violence alone compared to physical violence corroborated with the previous studies from India and abroad [16, 24]. Other studies have also demonstrated that sexual IPV alone was the strongest predictor of unintended pregnancies among all forms of IPV [25].

Chances of unintended pregnancies were significantly higher in specific populational subgroups such as younger age and women with multi-parity. These factors have also been highlighted in previous studies [26, 27]. Women in the age group of 15–24 years were more likely to have an unintended pregnancy as compared to older women, similar to a previous report [28]. Families where very young girls are married may hold traditional masculine ideologies. There is little resistance against sexual IPV, and reproductive rights are also compromised [29]. Though the prevalence of sexual IPV increases with the age of the women, protection against unintended pregnancies can be attributed to better knowledge and control over contraceptives and the desire to start a family in middle age groups [30]. Previous studies have also shown parity as a predictor of unintended pregnancy [31, 32]. We argue that women who have never had a child may desire children and, whereas those who may already have had their desired number of children can consider some pregnancies as unwanted. More unintended pregnancies in multiparous women are attributed to the loss of women’s reproductive autonomy that increases unwanted conception [33]. This also highlights the unmet need for access to safe abortion services [34].

There was a higher prevalence of unintended pregnancies among uneducated and poor women, but they did not emerge significantly in the regression analysis. However, education has been effective in reducing both mistimed and unwanted pregnancies in Sub-Saharan Africa through increased knowledge and use of sexual and reproductive health information and services [35]. Similarly, the regression findings related to wealth status deviate from other studies that have depicted wealth as a protective factor. Wealth influences access to reproductive health knowledge and facilitates participation in the social networks that support family planning and help attain a reduction infertility [36]. We also observed a high prevalence of unintended pregnancies in rural areas, among Muslims and women from Scheduled caste and tribes, but they were also not observed significantly in the regression analysis. According to the urban/rural distribution, a separate analysis among currently pregnant women from Nepal also reported no significant difference in the likelihood of unintended pregnancy according to the urban/rural distribution [36]. Previous studies have also depicted higher prevalence in Muslim women. This might be because of differences in doctrines among the women along the lines of religion [37]. Belonging to the scheduled tribe increased the chances of unintended pregnancy, similar to the findings from NFHS-3 [38, 39].

The present research is strengthened by the use of nationally representative datasets that have been collected using a standardized methodology, making the findings more reliable and comparable to other countries. The variables used for model building were chosen following a rigorous literature review. Finally, the use of the two step regression model based on a theoretical approach to predict the risk factors, supplemented our efforts to identify the direct and indirect ways in which these factors contribute to the development of unintended pregnancies and further clarify the inconsistent findings of previous studies.

However, certain limitations should be acknowledged that may arise because of the cross-sectional nature of the data collection. Although the temporality of events suggests a causal influence of sexual IPV on unintended pregnancy, inferences regarding causation must be drawn cautiously. To have more robust evidence a, longitudinal analysis or access to cross-sectional data that shows the timing of the event would be much preferred. At the same time, sexual IPV and pregnancy are very personal topics to discuss in a country like India and are neglected due to the discomfort about acknowledging IPV. Sexual IPV is self-reported, and the results may underestimate the overall problem in the country. Reporting may also vary according to the socio-economic characteristics of the women. Also, there is little information ﻿to compare the women’s reported intentions during or after pregnancy and their pre-pregnancy intentions. Finally, we could not include early miscarriages and abortions﻿, and they may add to the estimated burden.

## Conclusions and policy implications

The study intends to examine the effect of sexual IPV on unintended pregnancies in India. We observed that sexual IPV has a significant role in unintended pregnancies. Keeping in mind the taboo associated with sexual IPV, women prefer not to disclose it. Neither do they have an option to acknowledge the unintended pregnancy that they are carrying and continue with a process that takes a toll on their physical and mental health. Therefore, effective means of counseling should be rolled out, keeping in mind the significant factors that can predict unintended pregnancies and are highlighted in our study. The Reproductive, Maternal, Child, and Adolescent Health Program (RMNCH+A) launched by the government of India in 2012 should thrust more on gender-based violence prevention through its health education component and focus on adolescent girls and women in reproductive age groups.

Nevertheless, the male should not be forgotten during these counseling sessions and should be taught to respect the needs and desires of their female counterparts. It is also essential to encourage women to adopt family planning methods regularly to control their parity. However, spouses should be sensitized to come forward and respect their female counterparts as avoiding violence is not the sole responsibility of women. We need interventions focusing on changing social norms at the contextual level to make this possible so that women are empowered to make informed decisions.

## Data Availability

Data and materials are freely available upon making an official request to the DHS team through the DHS website at https://dhsprogram.com/what-wedo/survey-Types/dHs.cfm
